# Leitfaden „Geschlechtergerechte Sprache in der DGRh e. V.“

**DOI:** 10.1007/s00393-022-01268-4

**Published:** 2022-09-16

**Authors:** Isabell Haase, Sarah Ohrndorf, Xenofon Baraliakos, Eugen Feist, Bimba Hoyer, Uta Kiltz, Michaela Köhm, Martin Krusche, Philipp Sewerin, Anna Voormann, Johanna Mucke

**Affiliations:** 1 Kommission für Chancengleichheit in der Rheumatologie der DGRh e. V., Berlin, Deutschland; 2 Poliklinik, Funktionsbereich und Hiller-Forschungszentrum für Rheumatologie, Universitätsklinikum Düsseldorf, Heinrich-Heine-Universität Düsseldorf, Düsseldorf, Deutschland; 3 Med. Klinik mit Schwerpunkt Rheumatologie und Klinische Immunologie, Charité – Universitätsmedizin Berlin, Berlin, Deutschland; 4 Rheumazentrum Ruhrgebiet Herne, Ruhr-Universität Bochum, Bochum, Deutschland; 5 Helios Fachklinik für Rheumatologie, Vogelsang-Gommern, Deutschland; 6 Klinik für Innere Medizin I, Sektion Rheumatologie und klinische Immunologie, Exzellenzzentrum Entzündungsmedizin, Universitätsklinikum Schleswig-Holstein, Campus Kiel, Kiel, Deutschland; 7 Rheumatologie, Universitätsklinikum Frankfurt & Fraunhofer Institut für Translationale Medizin und Pharmakologie ITMP, Frankfurt, Deutschland; 8 Sektion für Rheumatologie und Entzündliche Systemerkrankungen, Universitätsklinikum Hamburg-Eppendorf (UKE), Hamburg-Eppendorf, Deutschland; 9 Deutsche Gesellschaft für Rheumatologie, Berlin, Deutschland

Als Abbild unseres Denkens und gesellschaftlicher Strukturen unterliegt unsere Sprache einem kontinuierlichen Wandel. Ziel sollte es sein, eine diskriminierungsfreie und inklusive Sprache zu nutzen, die Menschen jeden Geschlechts wertschätzend anspricht. Eine geschlechtergerechte Sprache dient dazu, alle Geschlechter gleichermaßen sichtbar zu machen und in unser Bewusstsein zu rücken.

Die Rheumatologie ist vielfältig und der Zukunft zugewandt. Sie bietet für alle Geschlechter Entfaltungsmöglichkeiten. Dies sollten wir bewusst nach außen tragen, uns durch die Verwendung geschlechtergerechter Sprache offen für Entwicklungen und Veränderungen der Gesellschaft zeigen und uns als moderne, diverse und inklusive Fachgesellschaft präsentieren. Auch dem Nachwuchsmangel in unserem Fach können wir begegnen, indem wir gezielt Menschen aller Geschlechter willkommen heißen.

Eine geschlechtergerechte Sprache dient darüber hinaus dazu, der Diversität der Patient:innen gerecht zu werden und Frauen und diverse Personen nicht nur „mitzumeinen“, sondern gezielt zu adressieren.

Denn „Mitmeinen“ verfehlt das Ziel, unsichtbar machende Strukturen in Sprache und Denken abzubauen, spricht es doch in erster Linie Personen männlicher Geschlechtsidentität an. Untersuchungen haben zudem gezeigt, dass sich andere Geschlechter durch das generische Maskulinum schlicht weniger angesprochen oder „mitgemeint“ fühlen [1].

Der Einsatz einer geschlechtergerechten Sprache bedeutet für viele Nutzende sicherlich eine Umstellung und stellt bisweilen auch eine Herausforderung dar: So ist sie sperrig, stört den Lesefluss und ist bisher nicht in der deutschen Grammatik verankert. Viele Argumente sprechen scheinbar gegen sie. Ihr Ziel einer Antidiskriminierung und Gleichstellung der Geschlechter überwiegt unserer Meinung nach jedoch alle Widrigkeiten.

Der Rat für deutsche Rechtschreibung empfiehlt die Aufnahme einer konkreten geschlechtergerechten verkürzten Schreibform in das amtliche Regelwerk der deutschen Rechtschreibung zwar bislang nicht, betont jedoch, *„… dass allen Menschen mit geschlechtergerechter Sprache begegnet werden soll und sie sensibel angesprochen werden sollen“ *[2]. Sprache, insbesondere eine geschlechtergerechte Sprache, unterliegt einem raschen und stetigen Wandel, feste Regeln existieren bisher nicht. Bei diesem Leitfaden handelt es sich daher um eine Empfehlung, die kontinuierlich an die gesellschaftlichen und sprachlichen Entwicklungen angepasst werden wird. Wir unterstützen ihre Umsetzung in der Deutschen Gesellschaft für Rheumatologie e.V. (DGRh e. V.), es handelt sich jedoch nicht um eine Vorschrift und erhebt auch keinen absoluten Anspruch.

## Leitfaden

Die DGRh e. V. unterstützt einen Sprachgebrauch, der die Gleichstellung aller Geschlechter widerspiegelt.

Die folgenden Empfehlungen betreffen die interne und externe Kommunikation der DGRh e. V. Hierzu zählen interne Texte, interner und externer Mail- und Briefverkehr, Öffentlichkeitsarbeit und mündliche Beiträge mit öffentlichem Charakter (Pressestatements, Vorträge …).

Für die *Zeitschrift für Rheumatologie* gilt: Autor:innen dürfen frei über die Verwendung von geschlechtergerechter Sprache entscheiden. Dennoch sollten sie auf die Empfehlungen aufmerksam gemacht und der Leitfaden den Autor:innen zur Verfügung gestellt werden.

### Übergeordnete Grundsätze

- Das generische Maskulinum wird ausdrücklich nicht empfohlen.
- Verwendung geschlechtergerechter Sprache in interner und externer Kommunikation der DGRh.
- Zur Umsetzung empfehlen wir die Verwendung der folgenden Formulierungen in der angegebenen Reihenfolge:
  1. Geschlechtsneutrale Formulierungen
  2. Verwendung des Gender-Doppelpunktes
  3. Beidnennung
- Die Leserlichkeit sollte nicht über die Maßen eingeschränkt werden.

#### Konkrete Umsetzung

1. Geschlechtsneutrale Formulierungen/neutrale Substantivierung
  - Bspw. *Lehrkraft, Studierende, Behandelnde, Leitung, Auditorium statt Studenten, Leiter etc*.
  - Schließen dritte Geschlechtsoptionen mit ein
  - Sollten – wenn möglich – primär verwendet werden
2. Verwendung des Gender-Doppelpunktes
  - Bsp.: *Ein:e Patient:in*
  - Sollte die Leserlichkeit nicht behindern. Bei komplizierten Konstrukten besser zu geschlechterneutraler Formulierung oder Beidnennung wechseln
  - Cave: Nicht möglich, wenn die feminine Form mit Umlaut gebildet wird *(Ärztin/Arzt)*
3. Beidnennung (Paarform)
  - Bspw. *„Ärztinnen und Ärzte“*
  - Sollte die Leserlichkeit nicht behindern
  - Es ist üblich, aber nicht vorgeschrieben, die weibliche Form zuerst zu nennen.
  - Cave: Die Beidnennung schließt dritte Geschlechtsoptionen nicht mit ein
  - Auf eine verkürzte Beidnennung *(„Professor/-in“)* sollte zugunsten des Gender-Doppelpunktes verzichtet werden

## Sonstiges

- Komposita (Personen in zusammengesetzten Wörtern)
  - Wenn möglich umformulieren oder ersetzen
  - Bsp. *Fachärztliche Ausbildung *statt* Facharztausbildung*
- Pronomen
  - *„Alle“* statt *„jeder“*
  - Verwendung geschlechtsneutraler Pronomen wie *alle *oder* wer*
